# A phase II trial of durvalumab and tremelimumab in metastatic, non‐urothelial carcinoma of the urinary tract

**DOI:** 10.1002/cam4.3699

**Published:** 2020-12-31

**Authors:** Michal Sarfaty, Karissa Whiting, Min Yuen Teo, Chung‐Han Lee, Vanessa Peters, Jennifer Durocher, Ashley M. Regazzi, Asia S. McCoy, Grace Hettich, Achim A. Jungbluth, Hikmat Al‐Ahmadie, Irina Ostrovnaya, Joshua Chaim, Dean F. Bajorin, Jonathan E. Rosenberg, Gopa Iyer, Samuel A. Funt

**Affiliations:** ^1^ Genitourinary Oncology Service Department of Medicine Memorial Sloan Kettering Cancer Center New York NY USA; ^2^ Department of Epidemiology and Biostatistics Memorial Sloan Kettering Cancer Center New York NY USA; ^3^ Department of Pathology Memorial Sloan Kettering Cancer Center New York NY USA; ^4^ Department of Radiology Memorial Sloan Kettering Cancer Center New York NY USA; ^5^ Weill Cornell Medical College New York NY USA; ^6^ Davidoff Cancer Center Petah‐Tikva, Sackler Faculty of Medicine Tel‐Aviv University Tel‐Aviv Israel

**Keywords:** adenocarcinoma, immunotherapy, small cell carcinoma, squamous cell carcinoma, urothelial carcinoma, variant histology

## Abstract

**Background:**

Immune checkpoint blockade has made a significant impact on the clinical outcomes of patients with metastatic urothelial carcinoma (UC). However, evidence for this approach in patients with non‐UC of the urinary tract is limited.

**Methods:**

This was a phase II open‐label study of durvalumab 1500 mg and tremelimumab 75 mg every 4 weeks for four cycles followed by durvalumab 1500 mg every 4 weeks. Eligible patients had metastatic non‐UC with ECOG PS 0–1 regardless of prior therapy (except small cell carcinoma who were pretreated). The primary endpoint was overall response rate per RECIST v1.1. A Simon's minimax two‐stage design was employed, with 13 patients planned for stage one. Pre‐treatment tumors underwent PD‐L1 staining and next‐generation sequencing.

**Results:**

Thirteen patients were treated, including seven small cell carcinoma, three squamous cell carcinoma, and three adenocarcinoma. Eleven patients had visceral metastases. No responses were observed; 11 patients had PD and 2 patients had SD. Median PFS was 1.8 months (95% CI, 1.25‐not reached [NR]) with a median follow‐up of 7.38 months (range, 5.23–21.99 months). Median OS was 6.97 months (95% CI, 4.34‐NR). One patient's tumor was PD‐L1 positive and all sequenced tumors (*n* = 8) were microsatellite stable. Grades 3–4 treatment‐related adverse events occurred in 38.4% of patients.

**Conclusions:**

In a poor prognosis cohort of patients with non‐UC, durvalumab and tremelimumab lacked clinical activity while demonstrating a manageable safety profile.

## Graphical abstract

Metastatic, non‐UC tumors of the urinary tract have an aggressive clinical course and dismal prognosis. This phase II trial evaluating durvalumab and tremelimumab demonstrated a manageable safety profile but lack of clinical activity.

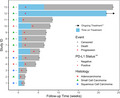

## 1 INTRODUCTION

The heterogeneity of tumors arising in the urothelial tract is reflected by the presence of divergent differentiation and variant morphologies.^1^, ^2^, ^3^ While two thirds of these cancers are classified as pure urothelial carcinoma (UC), the remaining third exhibit some element of non‐UC histology including squamous cell carcinoma (SCC), adenocarcinoma (ADC), and small cell/neuroendocrine carcinoma (NE).^4^ The SCC and ADC histologies (including those of urachal origin) are considered chemotherapy resistant, unlike NE, which is initially chemotherapy sensitive but almost uniformly progresses after treatment.^4^ Data to guide chemotherapy selection in patients with tumors containing pure/predominant non‐UC are limited with small single‐arm prospective trials in the first‐line, metastatic setting.^5^, ^6^ Otherwise, case reports and retrospective studies are available to inform management.^7^ Outcomes are typically poor,^8^, ^9^, ^10^ and new therapeutic strategies are urgently needed.

The development of immune checkpoint blockade (ICB) targeting the anti‐programed death 1 or anti‐programed death ligand 1 (PD‐1/L1) axis has made a significant impact on the clinical outcomes of patients with metastatic UC. These agents are FDA approved in the first‐line setting in cisplatin‐ineligible patients with PDL‐1 positive tumors, and in the platinum‐refractory setting, regardless of PD‐L1 staining.^11^ Overall response rates (ORR) in unselected patients are approximately 20%, with some patients experiencing dramatic and durable responses.^12^, ^13^ The FDA has also approved anti‐PD‐L1 therapy as maintenance among patients whose disease has not progressed with first‐line chemotherapy.^14^

Durvalumab is an engineered human IgG1 monoclonal antibody that blocks PD‐L1 and is approved for platinum‐refractory metastatic UC.^15^ In a phase I/II open‐label study of 191 patients with pure/predominant UC, durvalumab led to objective responses in 17.8% (95% CI, 12.7%–24.0%). Tremelimumab is a human anticytotoxic T lymphocyte‐associated antigen 4 (CTLA‐4), IgG class 2 monoclonal antibody that is being evaluated in combination with durvalumab in a variety of malignancies including UC. Combined ICB (cICB) of the PD‐1 and CTLA‐4 pathways with nivolumab and ipilimumab, respectively, leads to numerically higher objective response rates (ORRs) compared to PD‐1 blockade alone and is approved for use in several solid tumor types.^16^, ^17^, ^18^, ^19^

The clinical trials leading to regulatory approvals of ICB in patients with metastatic UC have excluded patients whose tumors had pure/predominant non‐UC. Nevertheless, there is significant rationale for this approach. cICB is active in both small cell and SCC of the lung.^20^, ^21^ Both anti‐PD‐L1 monotherapy as well as cICB have shown activity in small groups of patients with metastatic non‐UC.^22^, ^23^, ^24^ PD‐L1 expression by immunohistochemistry (IHC), which may enrich for response but does not preclude clinical benefit with anti‐PD‐1/L1 therapy, was shown to be higher in non‐UC than in classic/pure UC.^25^ PD‐L1 expression was also reported to be higher in SCC than in ADC,^26^ with no difference seen between UC with squamous differentiation and pure SCC.^27^ Finally, tumor mutation burden (TMB), which has also been associated with ICB response,^28^ was reported to be higher in NE tumors than in pure UC^,^.^29^ We hypothesized that the combination of durvalumab and tremelimumab would demonstrate clinical activity in metastatic non‐UC. Therefore, we initiated a study assessing the activity and safety of durvalumab and tremelimumab in this patient population.

## 2 PATIENTS AND METHODS

This phase II, open‐label study (ClinicalTrials.gov identifier, NCT03430895) was conducted at Memorial Sloan Kettering Cancer Center (MSKCC) in full accordance with the provisions of the Declaration of Helsinki and Good Clinical Practice Guidelines. The MSKCC institutional review board approved the study, and all patients provided written informed consent before participation.

### 2.1 Study design and population

This study enrolled patients with locally advanced and unresectable or metastatic non‐UC with measurable disease, according to the Response Evaluation Criteria in Solid Tumors (RECIST), version 1.1.^30^ Acceptable histologic subtypes included SCC, ADC, and NE. Patients with SCC and ADC were required to have a predominant (>50%) non‐UC component whereas, if any element of NE was present, the patients were classified as NE. Pathologic confirmation of non‐UC histology performed by a genitourinary pathologist (H.A) was required. All patients were required to have Eastern Cooperative Oncology Group performance status (PS) 0–1, a life expectancy of >12 weeks and adequate hematological, renal, and liver functions. Patients may have been previously untreated or may have progressed after any number of prior systemic therapies, except for patients with NE, who had to have progressed after at least one prior systemic therapy. Patients who received prior ICB were excluded.

The primary endpoint was ORR by RECIST v1.1. Secondary endpoints included progression‐free survival (PFS), overall survival (OS), duration of response, and safety and tolerability. An exploratory analysis included the association between PD‐L1 staining by IHC and response to therapy. Pretreatment tumors underwent next‐generation sequencing (NGS) to identify the predictors of response and resistance as well as to define the genomic landscape of non‐UC tumors.

### 2.2 Procedures

Patients received treatment with fixed‐dose durvalumab 1500 mg and tremelimumab 75 mg every 4 weeks for up to four cycles, and then durvalumab 1500 mg every 4 weeks starting 4 weeks after the last combination treatment for up to nine doses. All patients were treated until lack of clinical benefit, development of unacceptable toxicity, or completion of planned study treatment. Patients could continue treatment beyond progression if they met prespecified criteria for clinical benefit including stabilization/ improvement of disease‐related symptoms and no tumor growth at critical anatomic sites. Dose interruptions were allowed for toxicity; dose reductions were not permitted.

Clinical evaluation, complete blood cell counts, complete metabolic panel, and TSH were performed at baseline and on study. Cross‐sectional imaging was obtained at baseline and repeated every 8 weeks. Safety assessments were performed according to National Cancer Institute Common Terminology Criteria for Adverse Events (NCI CTCAE), version 4.0.

PD‐L1 expression was evaluated by IHC analysis in pretreatment tumor tissue using the SP‐263 anti‐PD‐L1 antibody assay (Ventana Medical Systems). PD‐L1 expression for both TC and IC in the tumor microenvironment was determined by the percentage of cells expressing membranous PD‐L1 staining.^31^ PD‐L1 high was defined as ≥25% of either tumor or immune cells staining for PD‐L1.^31^

Next‐generation sequencing using the Integrated Mutation Profiling of Actionable Cancer Targets platform was performed as previously described using DNA from pretreatment and matched normal specimens.^32^

### 2.3 Statistical analysis

The study was planned as a Simon's minimax two‐stage design, with 13 patients planned for stage one. If one or more responses were seen in stage one, an additional 14 patients were planned to be accrued for a total of 27. If no response was seen the study was planned to be terminated. This was based on a defined unacceptable ORR of 5% and acceptable rate of 20%, with 5% type I error, and 80% power. The response evaluable population was defined as all patients with a baseline disease assessment who have received at least one treatment with durvalumab and tremelimumab on study and have had either at least one post‐baseline disease assessment or withdrawn from study treatment prior to post‐baseline disease assessment due to clinical progression or death. Progression‐Free survival was defined as time from first treatment dose on study to first radiographic progression or death, whichever comes first. Overall survival was defined as time from first dose to death or last follow‐up. Event‐time distributions were analyzed using Kaplan–Meier curves. Descriptive statistics of TMB, select gene alterations, and prevalence of PD–L1 are presented.

## 3 RESULTS

### 3.1 Patient characteristics

From 20 March 2018 to 10 May 2019, 13 patients received treatment and were evaluable for the primary endpoint. Baseline patient and tumor characteristics are shown in Table 1 as well as Figure S1. The median age was 57 years (range 33–76) and 77% were males. The histology breakdown included seven bladder NE (54%), three bladder SCC (23%), and three ADC (23%, two urachal ADC and one primary bladder ADC). Eleven patients (85%) had visceral metastases, including seven patients (53%) with liver metastases. Eleven patients (85%) had received prior systemic therapy; of them, all NE patients were previously treated with etoposide and platinum‐based chemotherapy.

**TABLE 1:** Patient characteristics

Patient characteristics	Number (Range)	%
Age, years	57 (33–76)	
Male sex	10	76.9%
White race	11	84.6%
*Site of primary tumor*		
Bladder	10	76.9%
Upper tract	1	7.7%
Urachus	2	15.4%
*Histology*		
Pure small cell/NE	5	38.4%
UC with predominant small cell/NE features	2	15.4%
Pure squamous	1	7.7%
UC with predominant squamous differentiation	2	15.4%
Pure adenocarcinoma––Urachus	2	15.4%
Pure adenocarcinoma––Primary bladder	1	7.7%
*PD‐L1 status*		
High	1	7.7%
Low	10	76.9%
Unknown	2	15.4%
*ECOG performance status*		
0	4	30.8%
1	9	69.2%
*Metastatic sites at baseline*		
Visceral^a^	11	84.6%
Liver	7	53.8%
Lymph node / soft tissue only	2	15.4%
Previous cystectomy	5	38.5%
*Previous platinum‐based therapy*	11	84.6%
Cisplatin‐based	8	61.5%
Carboplatin‐based	2	15.4%
*Number of previous systemic regimens*		
0	2	15.4%
1	10	76.9%
2	1	7.7%
≥3	0	0.0%

Abbreviations: NE, neuroendocrine; UC, urothelial cell carcinoma.

^a^ Visceral metastasis defined as liver, lung, bone, or any non‐lymph node or soft tissue metastasis.

### 3.2 Efficacy

**FIGURE 1:**
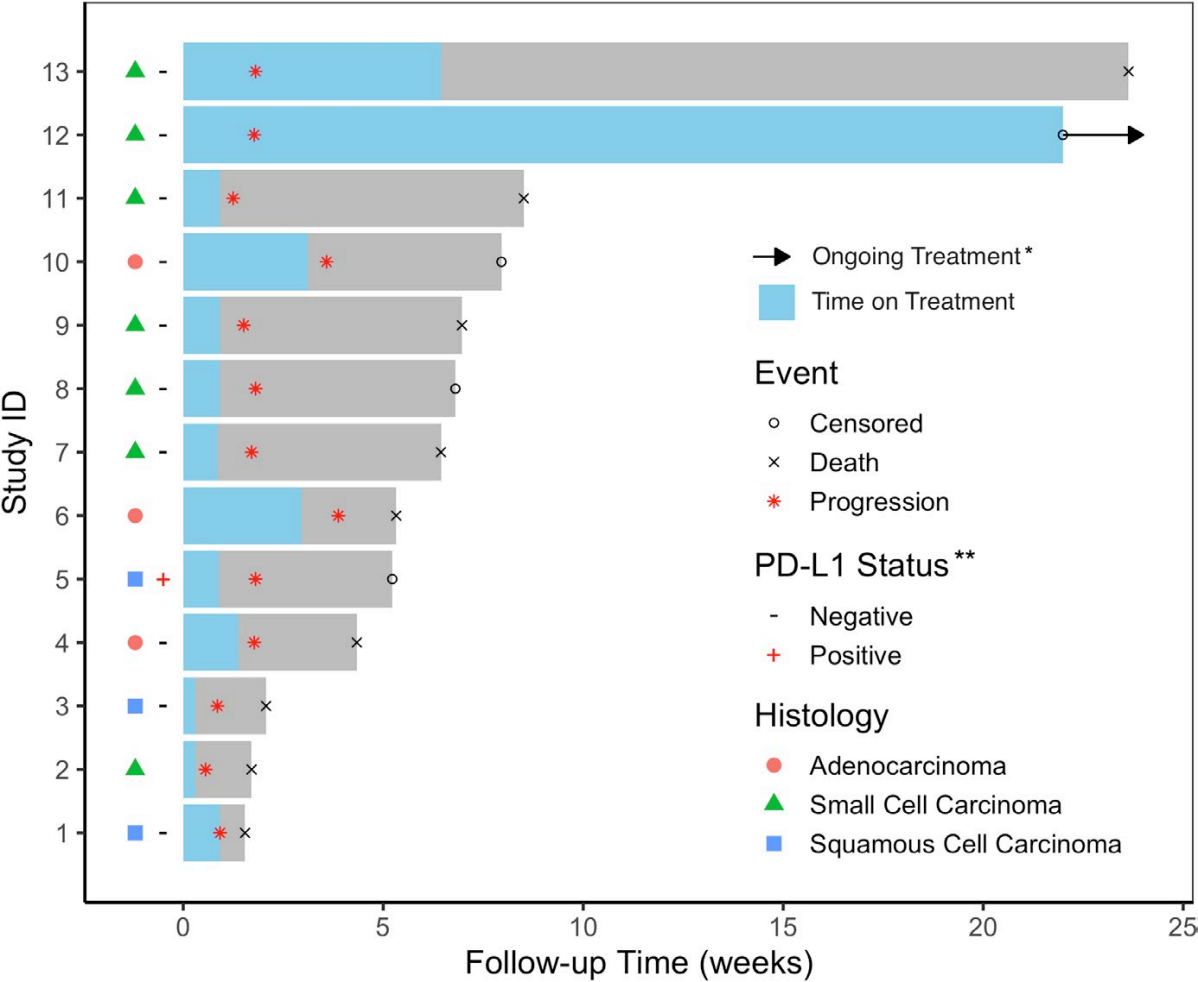
Swimmer's Plot. *Patient 12 completed 56 weeks of treatment on protocol and continues commercial use of durvalumab. * PD‐L1 status unknown for patients 2 and 6

**FIGURE 2:**
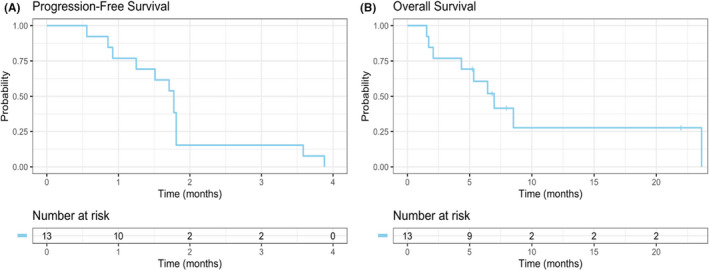
Progression‐free survival and overall survival

Patients received a median two cycles of therapy (range, 1–13). Stable disease (SD) was achieved in 2 patients (15%), and 11 patients (85%) had progressive disease as their best response (Figure 1). Median PFS was 1.8 months (Figure 2; 95% CI, 1.25‐not reached [NR]) with a median follow‐up (calculated among those alive) of 7.38 months (range, 5.23–21.99 months). Median OS was 6.97 months (Figure 2; 95% CI 95% CI, 4.34‐NR). At the time off the data‐cutoff (May 1, 2020), all patients had progressed and 9/13 (69.2%) had died (Figure 1). After a planned interim analysis for futility, the trial was terminated.

Of note, one patient with metastatic NE had mixed response on first disease assessment, with progressive nodal disease per RECIST criteria but shrinking liver metastasis with perceived clinical benefit. He developed brain metastases at cycle 5 that were treated with whole‐brain radiotherapy. As patient maintained his PS and imaging showed regression of his liver metastases, systemic treatment was continued. On repeat imaging, further reduction in his liver metastases was observed but an aortocaval node was irradiated during cycle 9 after growth caused pressure on the stomach. He eventually completed an additional four cycles of durvalumab and then, has continued off‐protocol, commercial use durvalumab 22 months after starting on study. His liver metastases have remained stable, and he has not required any additional local therapy.

### 3.3 PD‐L1 IHC and genomic data

**FIGURE 3:**
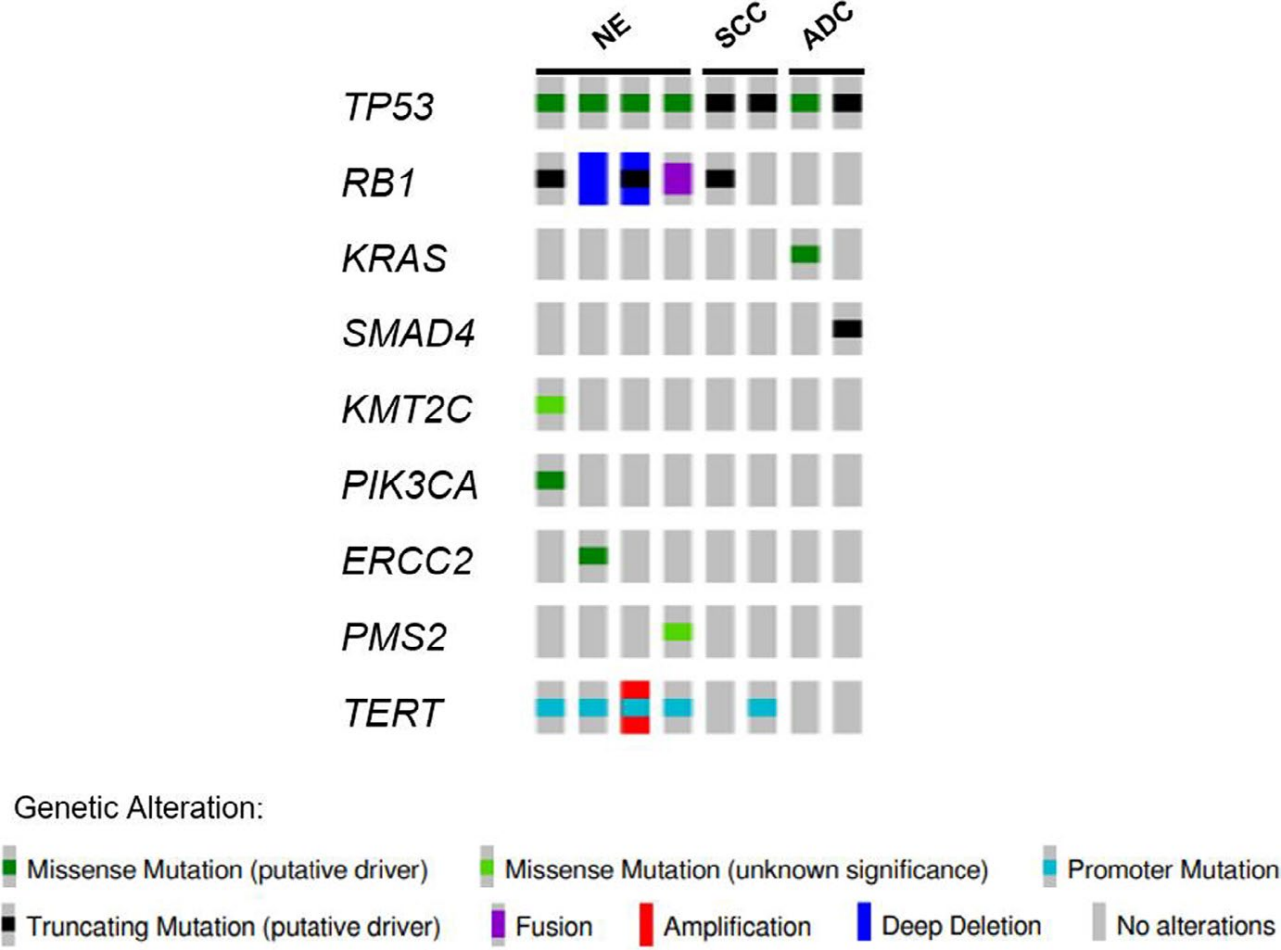
Oncoprint

Of the 11 patients whose tumors were successfully stained for PD‐L1, only 1 patient was positive (70% staining in tumor cells and 10% in immune cells). This patient developed disease progression after two cycles of treatment. MSK‐IMPACT was conducted on eight available tumors; of them, four were from the primary tumor and four from metastases; seven biopsies were taken prior to starting study treatment and one was taken at diagnosis, prior to previous line of treatment. All were microsatellite stable, the median TMB was 7.9 mut/Mb (range 3.5–13.2 mut/Mb), and the most frequent somatic alterations were identified in *TP53* (100%), *RB1*, and *TERT* (both 62%), see Figure 3 and Table S1. Of note, the tumor of the patient with NE histology who completed 13 months of protocol therapy with ongoing benefit from durvalumab monotherapy had the highest TMB (13.2 mutations/Mb), but was microsatellite stable and PD‐L1 negative (Figure 3).

### 3.4 Safety

All treated patients were evaluable for toxicity using CTCAEv4.0 criteria. Table 2 summarizes hematological and non‐hematological toxicities. Grades 3–4 treatment‐related adverse events occurred in 38.4% of patients, all on cICB treatment (colitis, *n* = 2; elevated amylase/lipase, *n* = 2; rash, *n* = 1; fatigue & muscle weakness, *n* = 1). Four patients required systemic steroids for treatment‐related adverse events (grade 3 rash, *n* = 1; grade 3 elevated lipase, *n* = 1; grade 3 colitis, *n* = 1; grade 2 dry mouth, anorexia, *n* = 1). One patient developed grade 3 colitis after discontinuing protocol therapy for progression of disease and required infliximab. Dose interruptions were required in four patients (30.7%), three of them due to toxicity. Progressive disease was the cause of treatment discontinuation for all patients.

**TABLE 2:** Treatment‐related adverse events

	Grade (*N* = 13)			
	1–2		3–4	
	No.	%	No.	%
Amylase increase	0	0	1	7.6
Lipase increase	0	0	1	7.6
Rash	3	23	1	7.6
Dry skin	2	15.3	0	0
Pruritus	1	7.6	0	0
Chills	1	7.6	0	0
Hot Flashes	1	7.6	0	0
Diarrhea	1	7.6	2	15.3
Constipation	1	7.6	0	0
Abdominal pain	1	7.6	0	0
Fatigue	7	53.8	1	7.6
Malaise	2	15.3	0	0
Arthralgia	2	15.3	0	0
Myalgia	2	15.3	0	0
Hypothyroidism	1	7.6	0	0
Headache	1	7.6	0	0
Nausea	1	7.6	0	0
Dysgeusia	1	7.6	0	0
Anorexia	3	23	0	0
Dry mouth	1	7.6	0	0
Peripheral sensory neuropathy	1	7.6	0	0
Eye disorder (Pain, Irritation)	1	7.6	0	0

## 4 DISCUSSION

This prospective study explosed cICB exclusively for the treatment of patients with metastatic, pure/predominant non‐UC. No objective responses were seen with durvalumab and tremelimumab. However, one patient completed a year of therapy with clinical benefit and reduction in liver metastases. Additionally, treatment was generally well tolerated with no new safety concerns outside of the known toxicity profile for ICB.^33^, ^34^

In contrast to our results, several small prospective studies have demonstrated activity with ICB in non‐UC. In the multinational SAUL study, 1004 patients with advanced urothelial cancer were treated with atezolizumab, a PD‐L1 inhibitor, including 49 patients with non‐UC. The ORR in this subset was 9%, compared to 13% (95% CI 11%–16%) in the entire group.^22^ A phase I trial evaluated cabozantinib combined with nivolumab, with or without ipilimumab in 54 patients with different genitourinary cancers.^23^ One out of four patients with urachal ADC had a PR, and two patients with SCC responded (one PR and one CR) to cabozantinib combined with nivolumab. The combination of ipilimumab, nivolumab, and cabozantinib is currently being assessed in the phase II ICONIC trial in rare genitourinary tumors, including non‐UC patients.^35^ Finally, a phase II study of nivolumab and ipilimumab demonstrated responses in 7 out of 19 patients (ORR 37%, 80% CI: 22%–54%), with responses observed in 2 of 3 patients with NE (one with CR), 1 of 4 patients with urachal ADC, 1 of 4 patients with non‐urachal ADC, and 2 of 6 patients with SCC.^36^

We explored the genomic profile of eight tumors for which adequate tissue was available. As observed in other neuroendocrine cohorts,^4^, ^29^ TP53 and RB1 were co‐altered in all four NE histology tumors, but other neuronal genes previously described in neuroendocrine‐like bladder tumors^37^, ^38^ were not present. Additionally, a KRAS activating mutation was seen in one urachal ADC and a SMAD4 truncating mutation in a second, both of which have been reported in cohorts of urinary tract ADC.^39^, ^40^ Notably, the one patient who derived clinical benefit on study had a NE carcinoma that exhibited a high TMB, which has been associated with sensitivity to checkpoint blockade in UC^28^

Given the published data indicating a correlation between DDR gene alterations and response to checkpoint blockade in UC,^41^ we also looked for a similar correlation within our non‐UC cohort. One patient with NE had an ERCC2 S44L mutation. This patient did not respond to therapy. A variant of unknown significance in the mismatch repair protein PMS2 was detected in the single patient with NE who completed protocol therapy with perceived clinical benefit. No other alterations were detected within genes involved in canonical DDR pathways in this cohort.

There are important limitations to the current study. First, this is a single‐center study with no comparator arm, a small sample size, and consisting of an admixture of different histologies. Due to the rarity of metastatic, pure/predominant non‐UC, clinical trials are difficult to accrue and thus often group different variants, although their underlying biology is likely markedly different.^4^ A retrospective study assessing response to CPI in urothelial tract tumors showed similar ORR between non‐UC to UC, but worse survival for NE tumors.^42^ Our trial included a predominance of patients with NE tumors, making it difficult to exclude activity in patients with SCC and ADC. Second, we enrolled a poor prognosis population as evidenced by rapid clinical deterioration. All but two patients had visceral metastases and over half had liver metastases, which are independent, poor prognostic factors.^43^, ^44^ The percentage of patients with visceral and/or liver metastases in the trials evaluating ICB in patients with non‐UC has not been reported.^22^, ^23^, ^24^ Third, only one patient's tumor tested positive for PD‐L1. In the phase I/II CheckMate032 study assessing nivolumab with and without ipilimumab at for patients with metastatic UC,^45^ the benefit for combination nivolumab and ipilimumab was greater for patients with PD‐L1 positive tumors than for those with PD‐L1 negative tumors (ORR 58.1% vs. 23.8% with nivolumab 1 mg/kg and ipilimumab 3 mg/kg). Fourth, we have not included transcriptomic or peripheral blood analyses despite recent reports that TGFβ mediates resistance to ICB in patients with mUC^46^ and circulating myeloid‐derived suppressor cells are higher in patients with SCC.^47^ However, this work is currently ongoing in this and other cohorts of patients with non‐UC. Lastly, although there was a higher response proportion seen with the higher ipilimumab dose in CheckMate032,^45^ leading to use of this higher dose in the ongoing CheckMate901 trial,^48^ the clinical relevance of tremelimumab dosing in patients with metastatic UC remains undefined. In patients with unresectable hepatocellular carcinoma, durvalumab with a single priming dose of tremelimumab 300 mg demonstrated greater efficacy than durvalumab with tremelimumab 75 mg for four doses and is being evaluated in a phase III study.^49^ The DANUBE phase III trial^50^ assessing durvalumab with tremelimumab 75 mg for four doses compared to standard‐of‐care chemotherapy in patients with untreated, metastatic UC did not demonstrate improved OS in the intention‐to‐treat population, but a trend for improved survival in the high PD‐L1 subgroup was noted. Although this was a negative trial, further investigation of durvalumab with a single priming dose of tremelimumab 300 mg is warranted, as this regimen is potentially more efficacious.

The treatment landscape for patients with metastatic UC has rapidly evolved with the approval not only of five anti‐PD‐1/L1 inhibitors but also of erdafitinib,^51^ an FGFR1‐3 tyrosine kinase inhibitor, and enfortumab vedotin,^52^ an antibody‐drug conjugate targeting nectin‐4. Despite these remarkable advancements, there is still a paucity of data to guide the management of patients with pure/predominant non‐UC. Although our study evaluating durvalumab and tremelimumab was negative, these findings should not preclude future studies of ICB in these patients. Indeed, the field highly anticipates the publication of other completed or ongoing studies (Table 3) and encourages the evaluation of novel agents such as enfortumab vedotin for the management of pure/predominant non‐UC. Finally, there must be coordinated efforts to dissect the genomic landscape and tumor microenvironment of non‐UC tumors to inform a rational approach to management.

**TABLE 3:** Summary of ongoing CPI clinical trials for metastatic non‐UC

Identifier	Intervention	Phase	Start date	Population	# of pts	1° end point	Status
NCT03582475^53^	Pembrolizumab with Platinum‐Based Chemotherapy	Ib	12/20/2018	Small Cell/Neuroendocrine Cancers of Urothelium and Prostate	30	Safety, ORR, and DOR	Recruiting
NCT02834013^54^	Nivolumab and Ipilimumab	II	1/13/2017	Rare genitourinary tumors, including non‐UC	818	ORR	Recruiting
NCT03866382^35^	Ipilimumab, Cabozantinib, and Nivolumab	II	4/12/2019	Rare genitourinary tumors, including non‐UC	186	ORR	Recruiting
NCT03084471^55^	Durvalumab +/‐tremelimumab	IIIb	4/17/2017	Advanced Solid Malignancies, including non‐UC.	868	Safety	Active, not recruiting

Abbreviations: and NE, small cell/neuroendocrine carcinomaCR, Complete response; DOR, Duration of response; ORR, Overall/objective response rate; PR, Partial response; SCC, squamous cell carcinoma; UC, urothelial carcinoma, ADC, adenocarcinoma.

## 5 CONCLUSION

In a poor prognosis cohort of patients with metastatic, non‐UC, durvalumab, and tremelimumab lacked clinical activity while demonstrating a manageable safety profile. Novel treatments are urgently needed for patients with metastatic non‐UC.

## CONFLICT OF INTEREST

Michal Sarfaty reports consulting fees from Merck, Novartis, and Roche. Chung‐Han Lee reports institutional research funds from Bristol‐Myers Squib, Calithera, Eisai, Eli Lilly, Exelixis, and Merck, Pfizer and consulting for Amgen, Bristol‐Myers Squib, Exelixis, Eisai, Merck, Pfizer, and EMD Serono. Dean F. Bajorin reports consulting fees from Bristol Myers Squibb, Merck, Genentech‐Roche, AstraZeneca, and Pfizer and institutional research support from Merck, Genentech‐Roche, AstraZeneca, Novartis, and Bristol‐Myers Squibb. Jonathan E. Rosenberg reports consulting fees from AstraZeneca, Bristol‐Myers Squibb, Merck, Roche, Genentech, Seattle Genetics, Astellas, Boehringer Ingelheim, Janssen, Pfizer, EMD Serono, GSK, and Mirati, and institutional research funding from Bayer, Astra Zeneca, and Seattle. Gopa Iyer reports consulting or advisory role for Bayer, Janssen, and Mirati Therapeutics; and has received research funding from Mirati Therapeutics, Novartis, Debiopharm Group, and Bayer. Hikmat Al‐Ahmadie reports consulting or advisory role for Bristol Myers Squibb, EMD Serono, AstraZeneca/MedImmune, and Janssen Biotech. Samuel A. Funt reports consulting fees from Merck; institutional research support from AstraZeneca and Genentech/Roche; stock and other ownership interest in Urogen, Allogene Therapeutics, Neogene Therapeutics, Kronos Bio, Iconovir, and Vida Ventures. The other authors made no disclosures.

## DATA SHARING

The data that support the findings of this study are available from the corresponding author upon reasonable request.

## Supporting information

Supplementary Material
