# Supplementary material for: A phase II trial of durvalumab and tremelimumab in metastatic, non‐urothelial carcinoma of the urinary tract
Source: Cancer Med. 2020 Dec 31;10(3):1074–83. doi: 10.1002/cam4.3699 (PMC7897955; doi:10.1002/cam4.3699)
Supplement: Supplementary file 1 — Supplementary Material [file CAM4-10-1074-s001.docx]

**Supplemental Figure 1: Representative patient hematoxylin and eosin (H&E) slides**

**
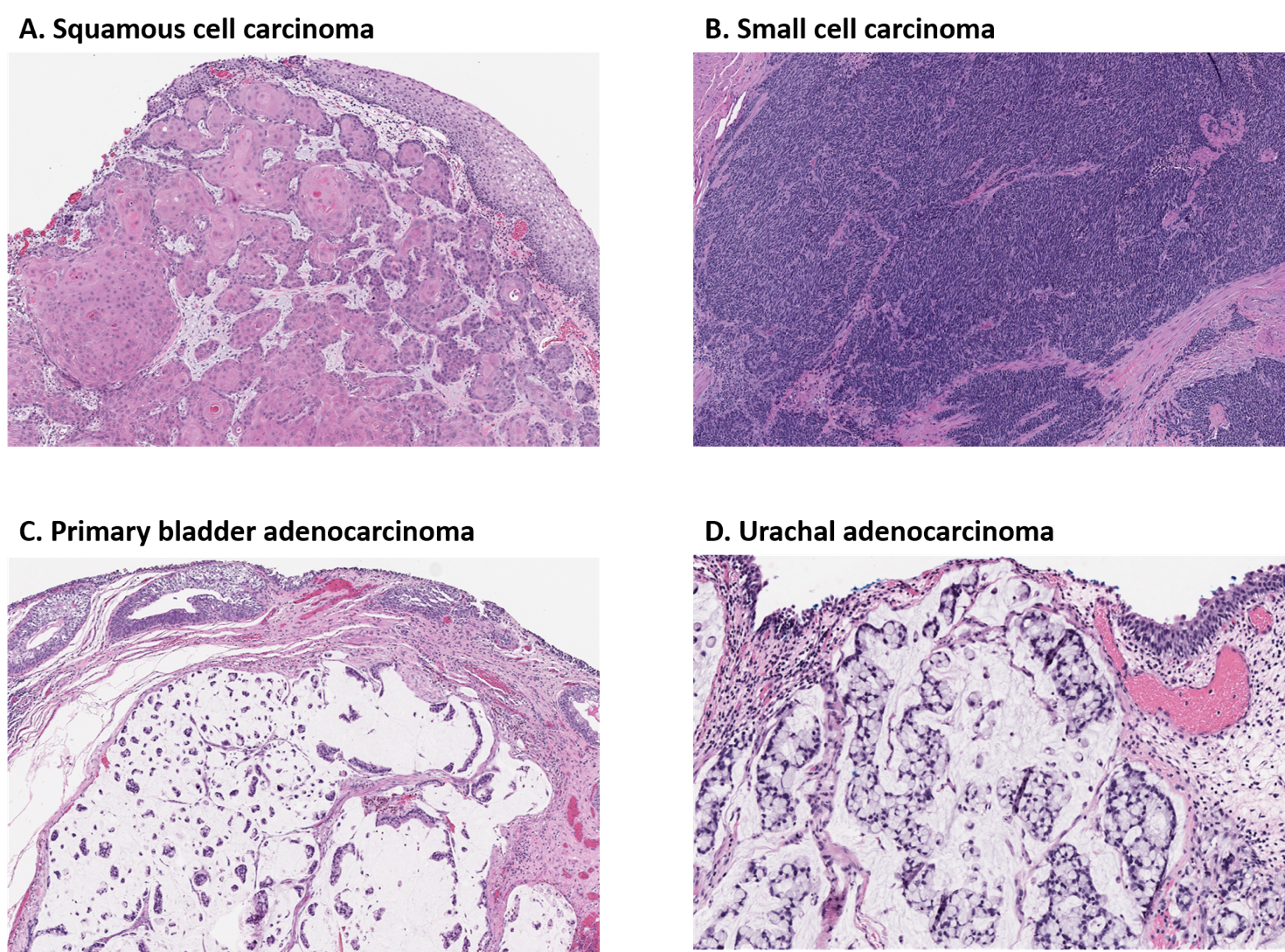
**

**Supplemental Table 1**

| **Study ID #** | **Histology** | **PDL-1 status** | **TMB** (Mut/Mb) | **MSI** |
| --- | --- | --- | --- | --- |
| **1** | Squamous cell | Negative | Unknown | Unknown |
| **2** | Small cell/ NE | Unknown | Unknown | Unknown |
| **3** | Squamous cell | Negative | 9.6 | Stable |
| **4** | Adenocarcinoma | Negative | Unknown | Unknown |
| **5** | Squamous cell | Positive | 4.4 | Stable |
| **6** | Adenocarcinoma | Unknown | 7.0 | Stable |
| **7** | Small cell/ NE | Negative | Unknown | Unknown |
| **8** | Small cell/ NE | Negative | 9.7 | Stable |
| **9** | Small cell/ NE | Negative | 8.8 | Stable |
| **10** | Adenocarcinoma | Negative | 3.5 | Stable |
| **11** | Small cell/ NE | Negative | Unknown | Unknown |
| **12** | Small cell/ NE | Negative | 13.2 | Stable |
| **13** | Small cell/ NE | Negative | 7 | Stable |
